# Comparison of estradiol hemihydrate 10 µg vaginal tablets versus estradiol hemihydrate 10 µg vaginal gel in postmenopausal women with vaginal atrophy: a randomized crossover study

**DOI:** 10.1007/s00404-025-08171-8

**Published:** 2025-10-17

**Authors:** Kanchanok Taemamu, Prasong Tanmahasamut, Manee Rattanachaiyanont, Thanyarat Wongwananuruk, Panicha Chantrapanichkul, Chatchai Areeswate

**Affiliations:** Department of Obstetrics and Gynecology, Faculty of Medicine Siriraj Hospital, Mahidol University, 2 Wanglang Road, Bangkoknoi, Bangkok, 10700 Thailand

**Keywords:** Estrogen replacement therapy, Menopause, Vaginal atrophy, Vaginal tablet, Vaginal gel

## Abstract

**Aim:**

This randomized crossover study evaluated the 12 week efficacy of a 10 µg estradiol hemihydrate vaginal tablet versus a vaginal gel (available at Siriraj Hospital, Thailand) in postmenopausal women with vaginal atrophy. Secondary endpoints included the most bothersome symptom, vaginal health index (VHI), vaginal pH, female sexual function index, serum estradiol, endometrial thickness, ease of use, comfort, and satisfaction.

**Methods:**

Ninety participants were randomized to receive either the 10 µg estradiol tablet or the gel daily for 2 weeks, followed by twice-weekly application for 10 weeks. Afterward, they switched to the alternate treatment for another 12 weeks using the same dosing regimen. Assessments of VHI, pH, vaginal maturation value (VMV), female sexual function index, endometrial thickness, and estradiol levels were conducted at baseline, 12 weeks, and 24 weeks.

**Results:**

Eighty-five participants completed the study. At 12 weeks (intention-to-treat analysis), the gel significantly increased VMV compared with the tablet (60.16 ± 12.00 vs 51.62 ± 23.77; P = 0.035; 95% CI 0.54 to 16.46), although the 95% CI included the noninferiority margin of 15. Per-protocol analysis showed no significant difference between groups. VHI improved, and pH decreased more markedly with the gel at 12 weeks. By 24 weeks, there were no significant between-group differences in VMV, pH, or most bothersome symptom. Acceptability was high for both treatments, although 55.3% of participants indicated a preference for continued gel use.

**Conclusions:**

Noninferiority of the 10 µg estradiol hemihydrate tablet relative to the gel could not be established. However, both treatments exhibited clinical benefits and high patient satisfaction, providing valuable insights for therapeutic decision-making in postmenopausal vaginal atrophy.

## Introduction

Genitourinary syndrome of menopause (GSM) typically manifests around 5 years after menopause, with symptoms intensifying with age if untreated [1]. The prevalence of GSM ranges from 27 to 84% among postmenopausal women [2, 3]. Common symptoms include vaginal dryness, dyspareunia, burning, dysuria, and recurrent urinary tract infections, which may occur singly or in combination [4]. The primary goal of treatment is to relieve symptoms associated with estrogen depletion and improve quality of life. An international survey found that 39‒51% of postmenopausal women worldwide experience vaginal symptoms [5]. In Thailand, one study reported that 51.7% of postmenopausal women experience vaginal dryness and 39.5% have painful intercourse [4].

Management options for GSM include hormonal and non-hormonal therapies [6]. Postmenopausal women with mild symptoms may benefit from lubricants and vaginal moisturizers, although these agents do not enhance vaginal tissue health [3, 6–8]. Vaginal estrogen is the most effective and safe treatment, owing to its low dose and minimal systemic absorption [9]. Multiple vaginal estrogen formulations exist worldwide, but in Thailand, local therapy has been limited to vaginal tablets. To offer a lower-cost alternative, Tanmahasamut and colleagues repurposed oral estrogen tablets to create a novel estradiol vaginal gel for Thai postmenopausal women [10]. A pilot study at Siriraj Hospital showed that this gel significantly reduced dryness and improved the vaginal maturation value (VMV) by an average of 41 ± 16.7 over 8 weeks compared with controls [10]. As a result, the gel was introduced at the Department of Obstetrics and Gynecology, Faculty of Medicine Siriraj Hospital, for women with GSM.

Femiest (Exeltis [Thailand] Co Ltd, Bangkok, Thailand), a 10 µg estradiol hemihydrate vaginal tablet derived from Vagifem (Novo Nordisk Healthcare AG, Bagsværd, Denmark), has been marketed in Thailand since 2022 and effectively alleviates GSM [11]. Another study showed that conjugated equine estrogen cream (1.25 mg) and a 25 µg 17β-estradiol vaginal tablet are similarly effective in reducing vaginal symptoms [12]. Most patients prefer tablet over cream formulations [12] although previous trials did not administer equivalent estrogen doses across formulations. Therefore, this study aimed to compare the efficacy and safety of two 10 µg 17β-estradiol hemihydrate vaginal preparations for treating postmenopausal vaginal atrophy at Siriraj Hospital.

## Methods

### Study design and setting

This 24 week randomized crossover study was conducted at the Gynecologic Endocrinology Unit, Department of Obstetrics and Gynecology, Faculty of Medicine Siriraj Hospital, Mahidol University, Bangkok, Thailand, from January to December 2024.

The study was carried out in accordance with the ethical principles of the 1964 Declaration of Helsinki and all subsequent amendments. The study protocol was approved by the Siriraj Institutional Review Board (reference Si-755/2023) on September 29, 2023. The study was registered with the Thai Clinical Trials Registry (TCTR 20231012006).

All participants were thoroughly informed about the study’s objectives, procedures, and potential risks. Written informed consent was obtained from every participant prior to enrollment.

### Inclusion criteria

Women were eligible if they were 45 years of age or older and had postmenopausal status, defined as either at least 12 months of amenorrhea or fewer than 12 months of amenorrhea with a serum follicle-stimulating hormone level exceeding 40 mIU/mL and an estradiol level below 30 pg/mL. Participants who had undergone bilateral oophorectomy and experienced moderate to severe postmenopausal vaginal symptoms—such as dryness, irritation, discomfort, discharge, or dyspareunia—were also included. Additional requirements were normal cervical cytology within the previous 3 years and an endometrial thickness of no more than 5 mm (by transvaginal ultrasonography) for women with a uterus.

### Exclusion criteria

The exclusion criteria included any known or suspected breast cancer, estrogen-dependent neoplasia, unexplained uterine bleeding, a history of thromboembolic disorders, cardiovascular disease, active liver disease, gallbladder disease, or genital tract infection. Women were also excluded if they had used hormones in the preceding 90 days, exhibited hypersensitivity to the study medication, or presented any contraindication to estrogen therapy.

### Drug preparation

The gel formulation was compounded using a base of K-Y Jelly, a hydrophilic medium that supports mucosal absorption. The pH of the compounded gel was approximately 3.5. No formal in vitro bioequivalence testing was conducted; however, previous study data showed its efficacy in improving VMV. Due to the small volume and local application, systemic absorption was minimal [10].

The estradiol hemihydrate gel was produced under pharmaceutical standards at the Department of Pharmacy, Siriraj Hospital, following the method described by Tanmahasamut et al [10]. The gel had a final volume of 0.8 mL and was packaged in a 3 mL disposable syringe equipped with a stopper (MultiCap; Mastertec GmbH & Co. KG, Oberhaid, Germany). According to stability testing [10], the gel remains stable for up to 24 weeks under refrigeration at 2‒8 °C and for up to 2 months at room temperature (30 °C).

The 10 µg estradiol hemihydrate vaginal tablet, Femiest, is a white, round, film-coated tablet with an approximate diameter of 6 mm [13]. Participants could administer the tablet with or without an applicator. When using an applicator, the user inserts it into the vagina until resistance is felt, presses the plunger to release the tablet, and removes the applicator. Tablets should be stored below 30 °C and should not be frozen [13].

### Study design and randomization

This 24 week crossover study randomized participants in a 1:1 ratio to receive either 10 µg of estradiol hemihydrate vaginal gel or 10 µg estradiol hemihydrate tablets. Computer-generated randomization codes were created using blocks of six. Forty-five women were assigned to the gel group, and 45 were assigned to the tablet group. The study drug for each participant was sealed in an opaque envelope marked with a corresponding code. A research nurse opened the envelopes sequentially and dispensed the appropriate medication.

### Intervention protocol

Participants initially used the assigned medication for 12 weeks. At the end of week 12, each group switched to the alternate formulation for the remaining 12 weeks. During the first 14 days of each 12-week period, participants self-administered the study medication intravaginally once daily, followed by twice-weekly administration (on Mondays and Thursdays) from week 3 to week 24. They were instructed to insert the medication between 9:00 PM and 10:00 PM, remain recumbent for 30 min post-insertion, and abstain from sexual activity on the day of administration.

### Study visits and assessments

At baseline, each participant’s medical history was recorded, including age, comorbidities, body weight, height, and years since menopause. Follow-up visits were performed at weeks 12 and 24. All evaluations were conducted by the same investigator (K.T.). Vaginal atrophy symptoms, the most bothersome symptom (MBS), vaginal pH, vaginal maturation index (VMI), VMV, vaginal health index (VHI), female sexual function index (FSFI), serum estradiol, endometrial thickness, adverse events, treatment compliance, and satisfaction were assessed at each visit. At the final assessment, participants indicated their preferred vaginal estrogen formulation.

### Outcome measures

The primary objective was to compare the mean change in VMV from baseline to week 12 between the estradiol hemihydrate vaginal tablet and gel groups. The secondary objectives included VMV changes from baseline to week 24, MBS scores, VHI, FSFI, vaginal pH, serum estradiol, and endometrial thickness. Additional aims were to evaluate ease, comfort, and satisfaction with drug use at 12 and 24 weeks following the crossover.

### Vaginal symptoms

The MBS was defined as the most severe symptom that adversely affected quality of life, with moderate to severe intensity [11, 14]. Each participant rated five possible symptoms—vaginal dryness, soreness, irritation, discharge, and dyspareunia—using a four-point scale: 0 (no symptom), 1 (mild), 2 (moderate), and 3 (severe). The MBS was then used to assess clinical improvement by evaluating changes in symptom severity over time.

### Vaginal health index

The VHI was determined following the methodology of the Robert Wood Johnson School of Medicine [15]. This assessment evaluates elasticity, fluid volume, vaginal pH, epithelium integrity, and moisture content. Each factor is scored on a scale of 1 to 5: 1 (none), 2 (poor), 3 (fair), 4 (good), and 5 (excellent). A single investigator (K.T.) performed these assessments for all participants.

### Vaginal cytology

Vaginal epithelial cells were collected from the junction of the upper and middle thirds of the lateral vaginal wall using an Ayre spatula. The Papanicolaou staining method involved placing the cells on a glass slide and fixing them with 95% alcohol. A single investigator (K.T.) obtained all samples, and a single cytologist (C.A.), both blinded to the treatment group, evaluated the slides.

The samples were utilized to ascertain the VMI and VMV. The VMI represents the percentage distribution of parabasal, intermediate, and superficial cells. The VMV is a single score (0‒100) that reflects the estrogenic status of the vaginal epithelium. It is calculated using the formula:

$${\text{VMV}}\, = \,\left( {0{\text{ x }}\% {\text{ parabasal cells}}} \right)\, + \,\left( {0.{\text{5 x }}\% {\text{ intermediate cells}}} \right)\, + \,({\text{1 x }}\% {\text{superficial cells}}).$$

A VMV of 0‒49 indicates minimal estrogen exposure, 50‒64 indicates a moderate estrogen effect, and 65‒100 indicates a high estrogen environment.

### Vaginal pH

Vaginal pH was measured immediately after speculum insertion, without lubrication, using a pH indicator strip ranging from 0 to 14.0 in 0.5 increments. The strip was applied to the upper third of the lateral vaginal wall, on the side opposite the site designated for the VMI sample.

### FSFI

Sexual function was evaluated using the Thai version of the FSFI [16, 17]. This translation underwent thorough testing to ensure accuracy and reliability [18]. Participants completed the questionnaire at baseline, week 12, and week 24. The FSFI assesses six domains—desire, arousal, lubrication, orgasm, overall satisfaction, and pain—through 19 questions scored on a scale of 0 to 5 or 1 to 5, depending on the question. Each domain score is multiplied by a specific coefficient and then summed to produce the total score. A total FSFI score below 26.5 is indicative of female sexual dysfunction.

### Sample size calculation

This study was designed as a noninferiority trial to compare the VMV at 12 weeks between 10 µg estradiol hemihydrate gel and 10 µg estradiol hemihydrate tablets in postmenopausal women with vaginal atrophy. The target noninferiority margin was set at 15, meaning that a mean difference in VMV below 15 would suggest equivalent treatment efficacy. The margin and estimated standard deviation [25] were guided by data from Tanmahasamut et al. [10] With a one-sided type I error of 0.05 and 80% power, the required sample size was calculated to be 35 participants per group. Allowing for a 20% dropout rate, 45 participants were needed in each group.

### Statistical analysis

Data were analyzed using IBM SPSS Statistics, version 29 (IBM Corp, Armonk, NY, USA). Categorical variables were described using frequencies and percentages, and comparisons were made with the chi-square test. Parametric continuous data were analyzed using Student’s t-test. Efficacy and safety parameters were assessed at each visit by the investigator and patients. The primary outcome (VMV) was evaluated via intention-to-treat analysis, which included all randomized participants who received at least one dose of medication. Missing data were handled using a worst-case approach, whereby the missing VMV was considered unchanged from baseline. VMV was also analyzed in the per-protocol population. Nonparametric data were evaluated with the Mann‒Whitney *U* test for between-group comparisons and the Friedman test for within-group comparisons. Continuous data across multiple time points were assessed by repeated-measures ANOVA. Values are presented as numbers and percentages, means with standard deviations, or medians with interquartile ranges. All tests were two-tailed, and a P value less than 0.05 was considered statistically significant.

## Results

### Study design and timeline

This trial used a six-month randomized crossover design divided into two 12 week treatment periods. The study was conducted over 12 months, from January to December 2024 Of the 165 women screened, 90 were randomly assigned in a 1:1 ratio to two groups. Group 1 (n = 45) received 10 µg estradiol vaginal tablets in the first 12 weeks and then switched to 10 µg vaginal gel for the following 12 weeks. Group 2 (n = 45) received the gel during the first 12-week period and then switched to the tablet formulation for the second period.

**Fig. 1:**
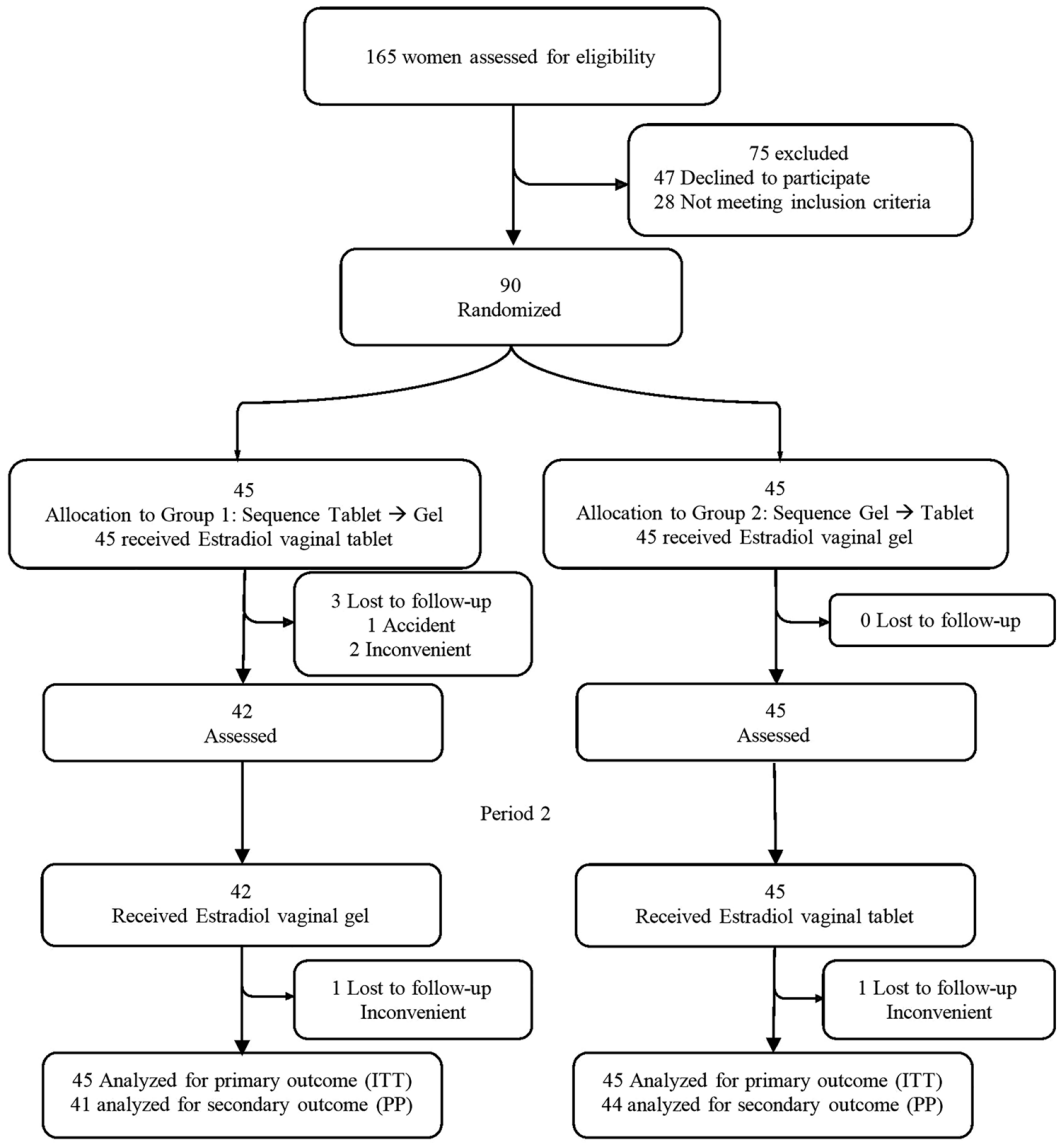
Patient disposition and analytical cohorts. This flow diagram illustrates the disposition of participants throught the study, including enrollment, randomization, allocation,follow-up, and analysis. The intention-to-treat and per-protocol (pp) populations are also indicated

The flow diagram at Fig. 1 illustrates the disposition of participants throughout the study, including enrollment, randomization, allocation, follow-up, and analysis.

### Discontinuations

During the first 12 week phase, three participants in Group 1 withdrew: two cited inconvenience in attending follow-up visits, and one encountered an accident. During the second treatment phase, two more participants withdrew (one from Group 1 and one from Group 2), both citing inconvenience.

### Rationale for no washout period

The absence of a washout period introduces the potential for a carryover effect; however, this approach was deliberate, as the optimal duration for complete drug clearance remains undefined. The primary endpoint was evaluated at 12 weeks, with the study design aiming to assess patient satisfaction under real-world conditions without the confounding influence of a medication-free interval.

### Baseline characteristics

Table 1 shows the baseline characteristics of both groups. The average age was 60.3 ± 6.5 years in Group 1 and 58.8 ± 6.7 years in Group 2 (P > 0.05). No significant differences were observed in body mass index, education, age at menopause, type of menopause, duration since menopause, or sexual status.

**Table 1:** Demographic and baseline characteristics of study participants

Characteristics	Group 1 (n = 45)	Group 2 (n = 45)	*P* value
Age (years)	60.29 ± 6.54	58.84 ± 6.71	0.304
Body mass index (kg/m^2^)	24.30 ± 3.67	23.89 ± 4.00	0.615
Education			
Undergraduate	25 (55.6%)	21 (46.7%)	0.201
Bachelor’s degree	18 (40.0%)	24 (53.3%)	
Master’s degree	2 (4.4%)	0 (0%)	
Age at menopause	48.89 ± 4.36	49.27 ± 4.59	0.690
Menopause			
Natural	39 (86.7%)	39 (86.7%)	1.000
Surgical	6 (13.3%)	6 (13.3%)	
Time since menopause			
< 5 years	7 (15.6%)	14 (31.1%)	0.197
5–10 years	18 (40%)	13 (28.9%)	
$>$10 years	20 (44.4%)	18 (40%)	
Hysterectomy	6 (13.3%)	6 (13.3%)	1.000
Parity			
Nulliparous	8 (17.8%)	10 (22.2%)	0.598
Multiparous	37 (82.2%)	35 (77.8%)	
Sexually active	17 (37.8%)	18 (40%)	0.829

Data are presented as mean with standard deviation, median (range), or n (%). Comparisons were made using Student’s *t* test for continuous variables and the chi-square test for categorical variables

**Fig. 2:**
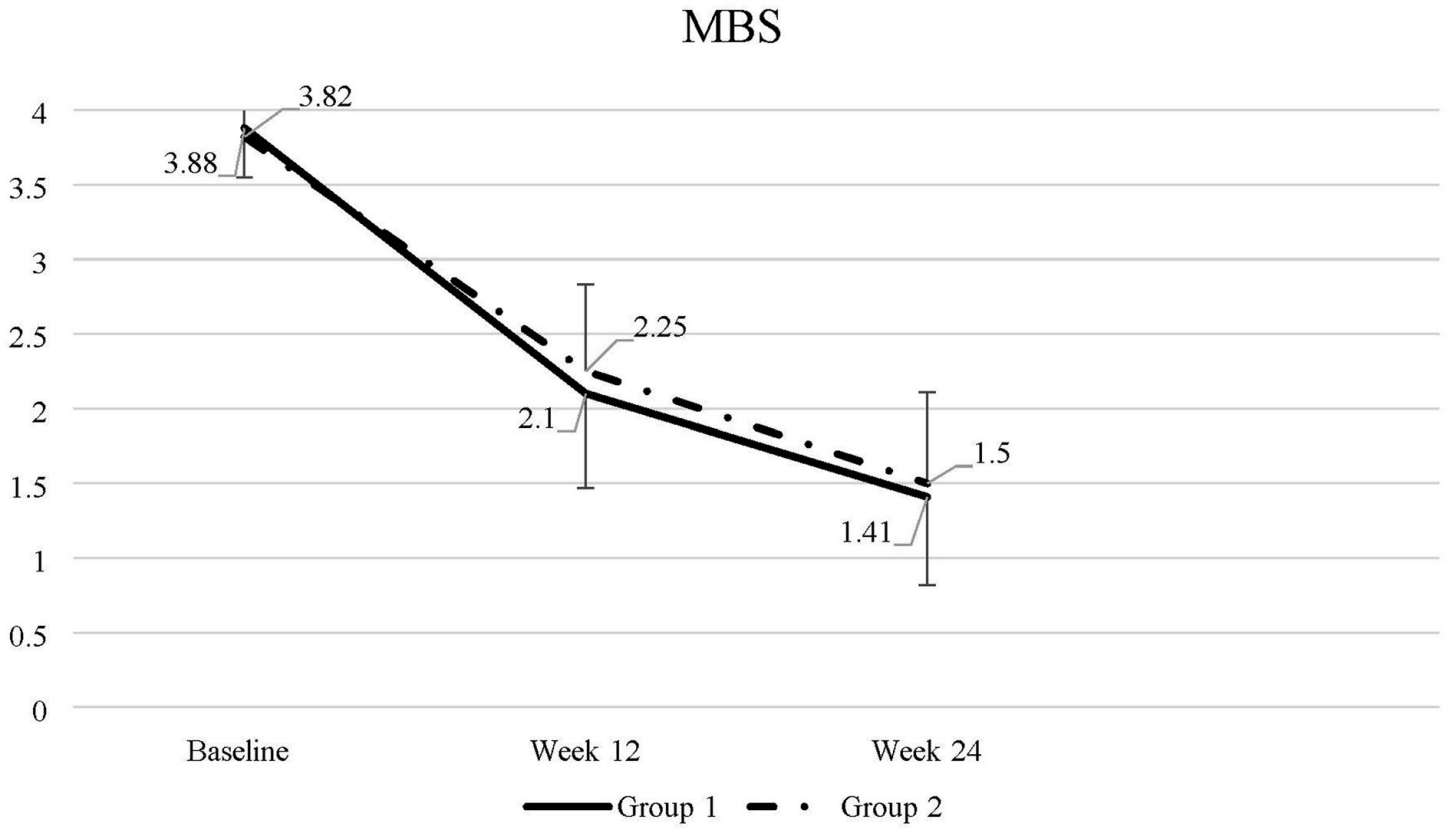
Changes in mean severity of the most bothersome symptom (MBS) over time. Data are presented as mean with standard deviation. Repeated-measures ANOVA was used to compare continuous data between and within groups. *P* < 0.001 compared to baseline. Group 1: tablet → gel; Group 2: gel → tablet

Baseline MBS data for both groups are presented in Table 2, and changes in mean MBS severity are illustrated in Fig. 2.

**Table 2:** Most bothersome symptom at baseline

Most bothersome symptom	Group 1 N = 45	Group 2 N = 45	*P* value
Dryness	28 (62.2%)	25 (55.6%)	0.929
Soreness	3 (6.7%)	4(8.9%)	
Irritation	1 (2.2%)	1 (2.2%)	
Discharge	0 (0%)	0 (0%)	
Dyspareunia	13 (28.9%)	15 (31.1%)	

Data are presented as n (%). Comparisons were made using the chi-square test for categorical variables

### VMI

Table 3 summarizes the VMI data. At baseline, parabasal cell counts were elevated in both groups, with a median of 59.0 (7.0, 91.5) in Group 1 and 39.0 (2.0, 82.0) in Group 2. By week 12, the median number of superficial cells had significantly increased in both groups: 16.5 (3.3, 35.3) in Group 1 (tablets) and 15.0 (8.0, 35.0) in Group 2 (gel; P < 0.01). The intermediate cells also showed a marked increase at week 12, with median values of 65.5 (52.0, 87.3) and 81.0 (64.0, 89.5), respectively (P < 0.01). No significant differences were detected between the two groups at this time point. After the crossover at the end of week 12, both groups continued to exhibit statistically significant increases in superficial and intermediate cells, along with decreases in parabasal cells, through week 24.

**Table 3:** Comparison of vaginal maturation index (VMI) between groups

VMI	Group 1	Group 2	*P* value
Parabasal cells			
Baseline	59.00 (7.00,91.50)	39.00 (2.00, 82.00)	0.215
Week 12	0.00 (0.00, 9.25)	0.00 (0.00, 1.50)	0.242
Week 24	0.00 (0.00, 7.00)	0.00 (0.00, 3.25)	0.165
*P* value	< 0.001	< 0.001	
Intermediate cells			
Baseline	37.00 (8.50, 75.00)	55.00 (18.00, 78.00)	0.279
Week 12	65.50 (52.00, 87.25)	81.00 (64.00, 89.50)	0.059
Week 24	77.00 (58.00, 89.00)	82.00 (71.75, 92.50)	0.063
*P* value	< 0.001	< 0.001	
Superficial cells			
Baseline	0.00 (0.00, 7.00)	0.00 (0.00, 10.00)	0.698
Week 12	16.50 (3.25, 35.25)	15.00 (8.00, 35.00)	0.734
Week 24	14.00 (3.50, 28.50)	10.00 (5.00, 20.00)	0.606
*P* value	< 0.001	< 0.001	

Data are presented as median (interquartile range). Comparisons were performed using the Mann–Whitney *U* test for between group analysis and the Friedman test for within-group analysis. Group 1: tablet → gel; Group 2: gel → tablet

### VMV

**Fig. 3:**
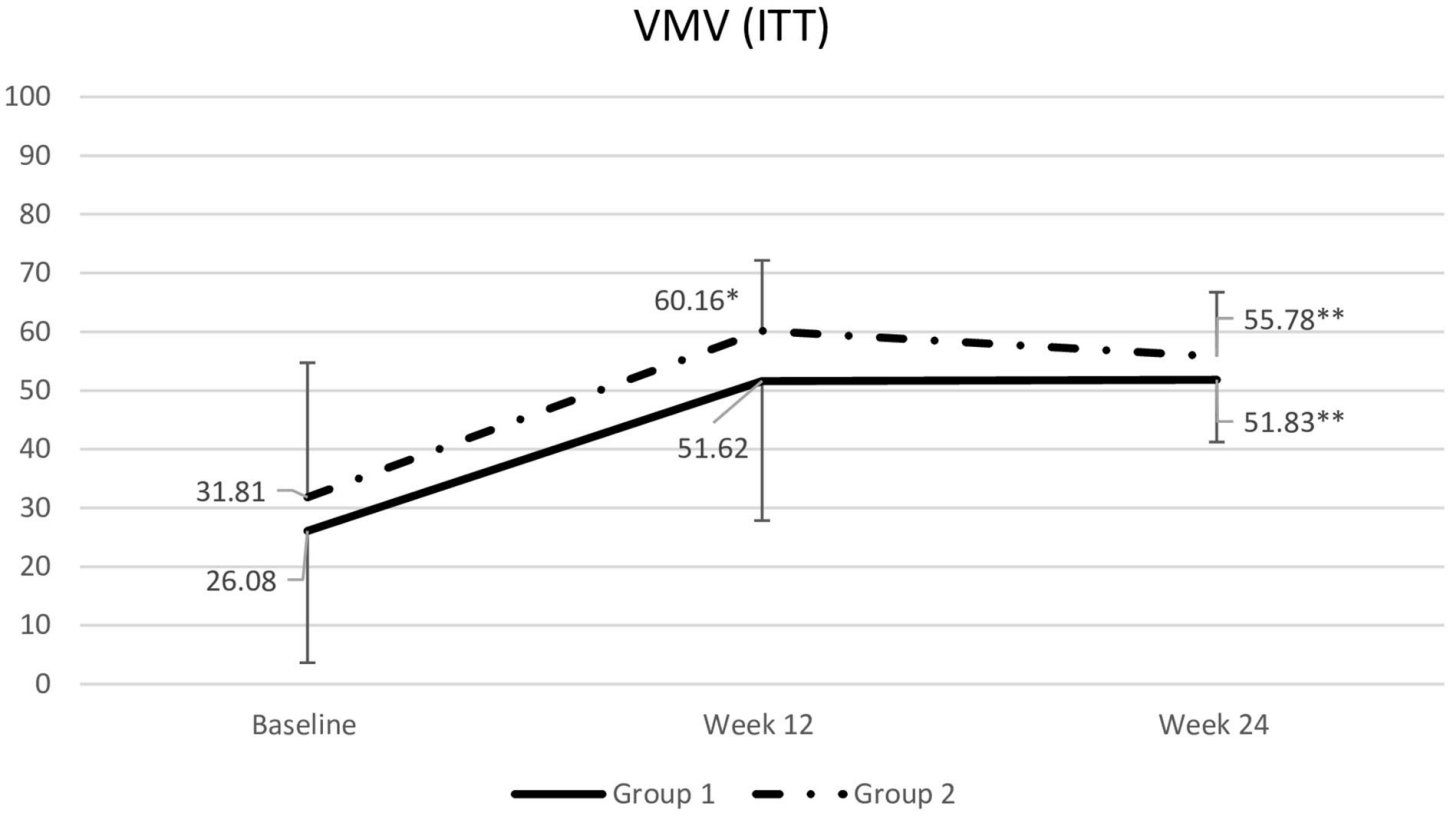
Comparison of vaginal maturation value (ITT). Data are mean ± standard deviation. Data were compared using repeated Measures ANOVA for continuous data between and within group. *p < .001 compared to baseline. **p > 0.05 compared to week 12

At week 12, intention-to-treat analysis showed that the estradiol hemihydrate vaginal gel increased VMV significantly more than the tablet (60.2 ± 12.0 vs 51.6 ± 23.8; P = 0.035, 95% CI 0.5 to 16.5). In the per-protocol analysis, both treatments raised VMV at week 12 (54.9 ± 21.0 vs 60.2 ± 12.0, respectively; P = 0.161, 95% CI ‒2.1 to 12.6). After switching treatments at the end of week 12, both groups maintained improvements from baseline through week 24, with no significant differences between the two groups (Fig. 3).

### VHI and pH

**Fig. 4:**
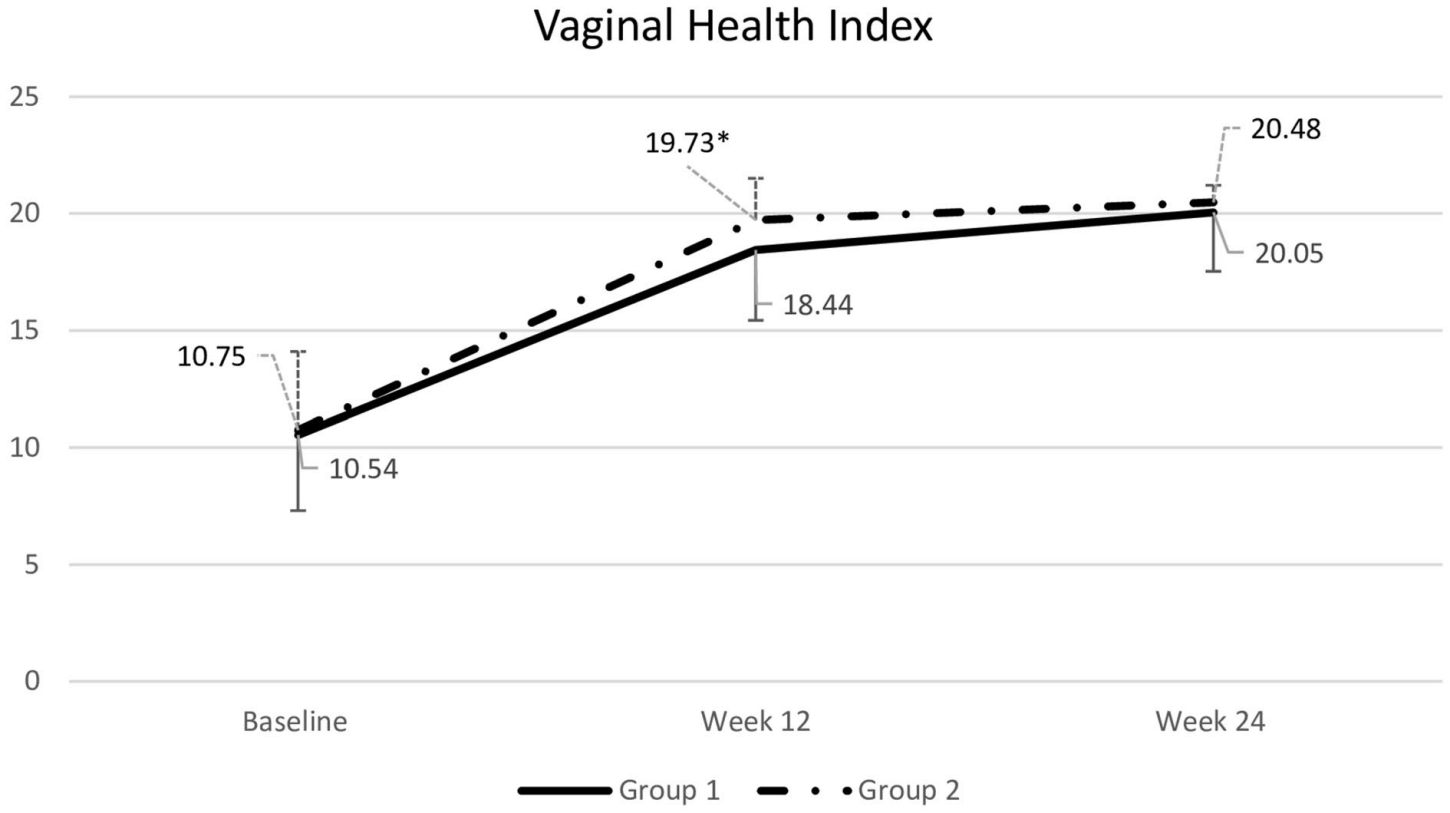
Comparsion of vaginal health index (VHI) between groups. Data are presented as mean with standard deviation. Repeated-measures ANOVA was used for comparisons. *p = 0.018 compared to Group 1. Group 1: tablet → gel; Group 2: gel → tablet

**Fig. 5:**
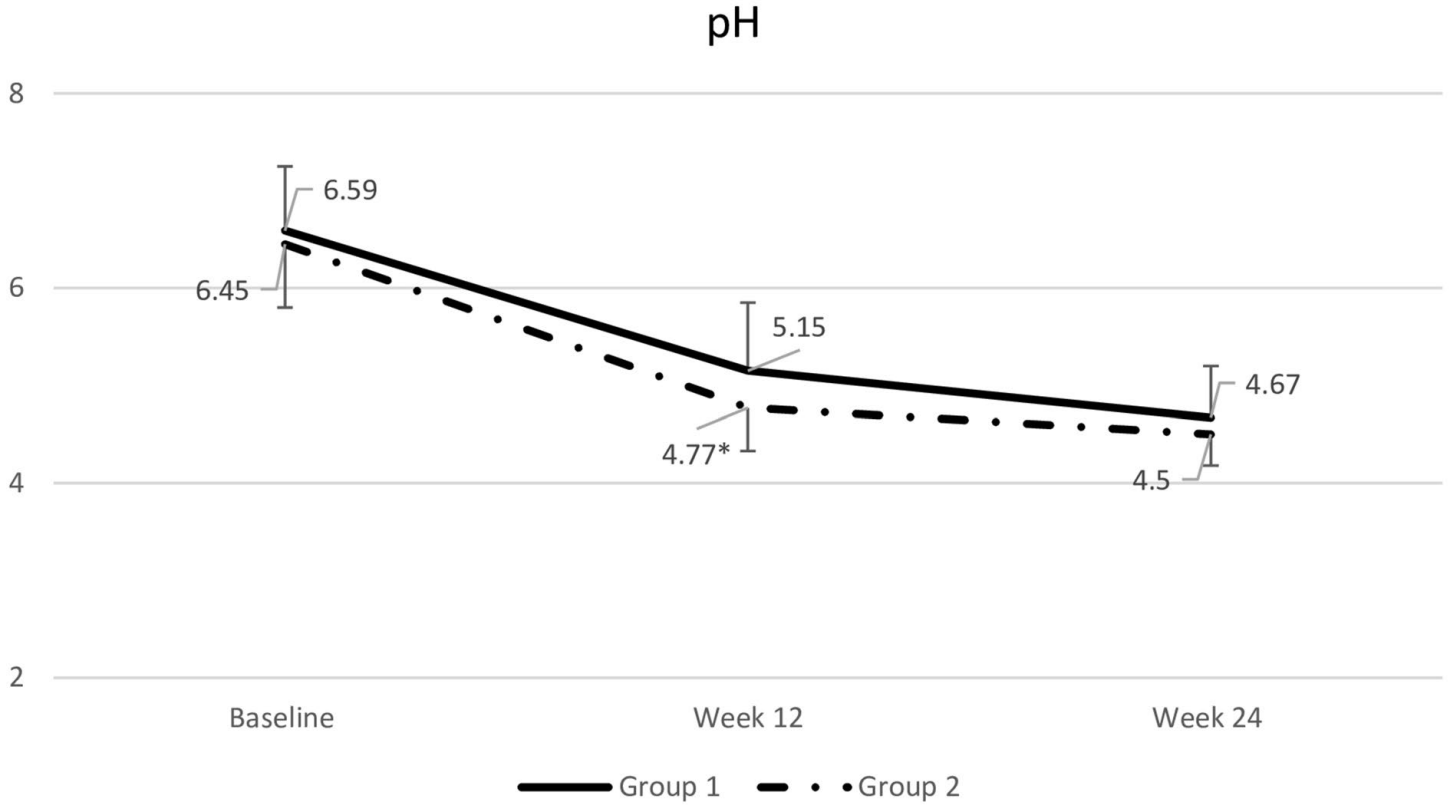
Comparsion of vaginal pH between groups. Data are presented as mean with standard deviation. Repeated-measures ANOVA was used for comparisons. *p = 0.018 compared to Group 1. Group 1: tablet → gel; Group 2: gel → tablet.

At week 12, participants receiving the gel had a significantly higher VHI than those receiving the tablet. However, by week 24, after the crossover, there was no significant difference in VHI between the two groups (Fig. 4). Similarly, vaginal pH at week 12 was significantly lower in the gel group than in the tablet group (Fig. 5).

### FSFI

Among the 90 participants, 35 (37.8%) were sexually active (17 in Group 1 and 18 in Group 2). Both groups showed nonsignificant increases in total FSFI scores following treatment. However, they demonstrated a statistically significant improvement in the pain domain at week 24 compared to baseline (Table 4). Group 1 showed a significant improvement in the arousal domain, whereas Group 2 showed a significant improvement in the satisfaction domain.

**Table 4:** Changes in female sexual function index (FSFI) domains over time

Comparison of change in domain of FSFI	Group 1 N = 17	Group 2 N = 18	Mean difference	95% CI of difference	*P* value
Total score					
Baseline	15.66 ± 6.83	15.54 ± 5.30	− 0.11	− 4.31 to 4.08	0.956
Week 12	18.05 ± 8.21	18.42 ± 6.25	0.36	− 4.63 to 5.36	0.883
Week 24	17.56 ± 8.85	18.82 ± 6.43	1.26	− 4.04 to 6.56	0.632
*P* value	0.271	0.071			
Desire					
Baseline	2.61 ± 1.19	2.33 ± 0.97	− 028	− 1.04 to 0.48	0.456
Week 12	2.75 ± 1.18	2.79 ± 0.82	− 0.035	− 0.68 to 0.75	0.920
Week 24	2.96 ± 1.25	2.75 ± 1.26	− 0.21	− 1.09 to 0.66	0.625
*P* value	0.403	0.097			
Arousal					
Baseline	2.38 ± 1.51	2.33 ± 1.13	− 0.05	− 0.99 to 0.88	0.909
Week 12	2.91 ± 1.76	2.75 ± 1.22	− 0.16	− 1.22 to 0.90	0.762
Week 24	3.26 ± 1.37	3.04 ± 1.34	− 0.23	− 1.18 to 0.72	0.626
*P* value	0.027	0.132			
Lubrication					
Baseline	2.31 ± 1.30	2.38 ± 1.01	0.07	− 0.75 to 0.89	0.861
Week 12	2.42 ± 1.43	2.37 ± 1.20	− 0.05	− 0.98 to 0.87	0.908
Week 24	2.26 ± 1.19	2.67 ± 0.97	0.40	0.35 to 1.17	0.284
*P* value	0.893	0.631		–	
Orgasm					
Baseline	2.68 ± 1.47	2.92 ± 1.23	0.24	− 0.71 to 1.18	0.616
Week 12	2.64 ± 1.61	2.87 ± 1.44	0.24	− 0.83 to 1.31	0.657
Week 24	2.94 ± 1.59	2.97 ± 1.54	0.24	− 1.06 to 1.11	0.965
*P* value	0.657	0.979			
Satisfaction					
Baseline	3.22 ± 1.58	3.04 ± 1.05	− 0.19	− 1.12 to 0.75	0.685
Week 12	3.67 ± 1.61	3.88 ± 1.07	0.21	− 0.74 to 1.17	0.654
Week 24	3.98 ± 1.43	3.81 ± 1.13	− 0.16	− 1.07 to 0.74	0.712
*P* value	0.053	0.016			
Pain					
Baseline	2.35 ± 1.47	2.80 ± 1.52	0.45	− 0.60 to 1.49	0.390
Week 12	1.94 ± 1.94	3.81 ± 1.9	0.87	− 0.50 to 2.24	0.206
Week 24	3.37 ± 1.90	3.67 ± 2.07	0.31	− 1.08 to 1.70	0.657
*P* value	0.043	0.032			

Data are presented as mean with standard deviation. Comparisons were made using repeated-measures ANOVA for within-group and between-group comparisons. Group 1: tablet → gel; Group 2: gel → tablet

### Endometrial thickness.

**Fig. 6:**
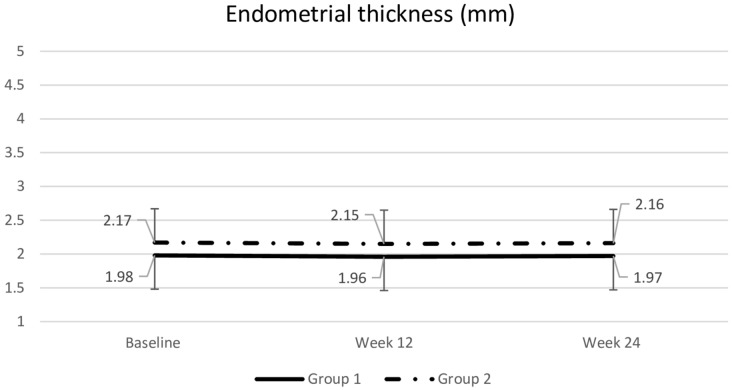
Comparsion of endometrial thickness between groups. Data are presented as mean with standard deviation. Repeated-measures ANOVA was used for continuous data comparisons between and within groups. *p > 0.05 compared with baseline and between treatment groups. Group 1: tablet → gel; Group 2: gel → tablet

At week 12, the mean endometrial thickness was similar in both groups: 2.0 ± 0.5 mm for the tablet users and 2.2 ± 0.6 mm for the gel users. Following the switch, no significant increase in endometrial thickness was noted at week 24 (Fig. 6).

### Serum estradiol levels

**Fig. 7:**
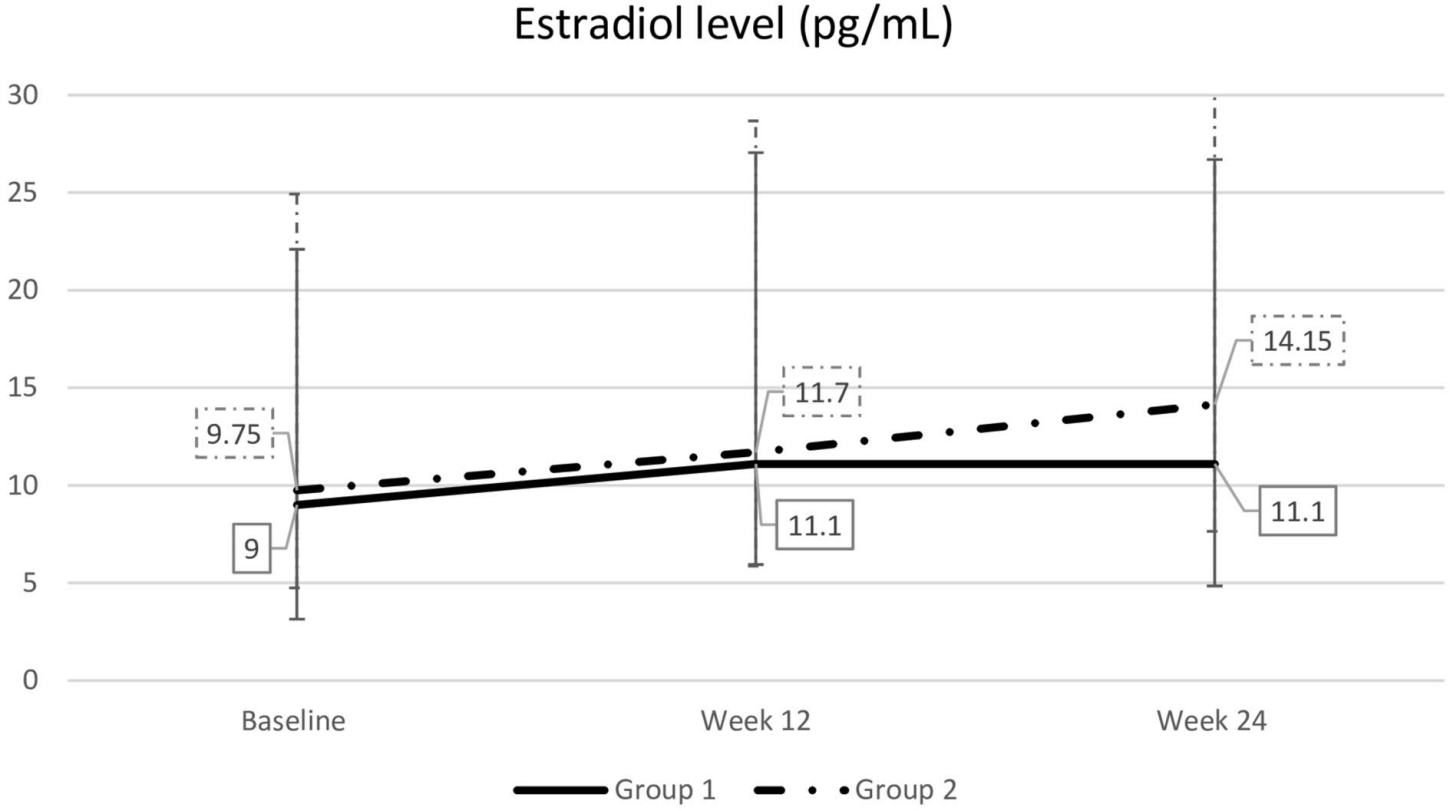
Comparsion of serum estradiol levels between groups. Data are presented as median (interquartile range). The Mann–Whitney *U* test was used for between-group comparisons, and the Friedman test was used for within-group comparisons. *p* > 0.05 compared with baseline and between treatment groups. Group 1: tablet → gel; Group 2: gel → tablet

Serum estradiol levels did not significantly increase at weeks 12 or 24 and remained comparable between both groups. Levels also remained within the normal postmenopausal range (Fig. 7).

### Adverse events

One participant experienced uterine bleeding after 2 months of tablet use. Physical examination revealed no cervical or vaginal lesions, and endometrial thickness measured 4.2 mm. Endometrial sampling confirmed an atrophic endometrium. Another participant reported abnormal vaginal bleeding after 10 weeks of tablet use. Examination revealed a vaginal abrasion on the left vaginal wall, with an endometrial thickness of 3.4 mm. Both participants continued in the study through completion.

In addition, a 51-year-old participant with 4 years of menopause was diagnosed with breast cancer during the study period. Her most recent annual mammogram 7 months prior had shown BI-RADS 3, and the examination following 6 weeks of estradiol tablet use revealed BI-RADS 4A. A core needle biopsy confirmed invasive ductal carcinoma. She subsequently underwent a nipple-sparing mastectomy, with pathology indicating a triple-negative invasive ductal carcinoma (estrogen receptor, progesterone receptor, and human epidermal growth factor receptor 2 all negative). After consulting with her surgeon—who did not advise discontinuing the trial medication—she chose to remain in the study for the full 24 weeks to address her severe vaginal symptoms. Throughout the study, her serum estradiol level remained within the postmenopausal range [19].

### Compliance

Compliance was high in both groups at weeks 12 and 24. At week 12, 95.2% of participants in the tablet group and 97.8% in the gel group adhered to the treatment protocol. At week 24, the adherence rates were 93.2% in the tablet group and 92.7% in the gel group.

### Participant preferences

**Fig. 8:**
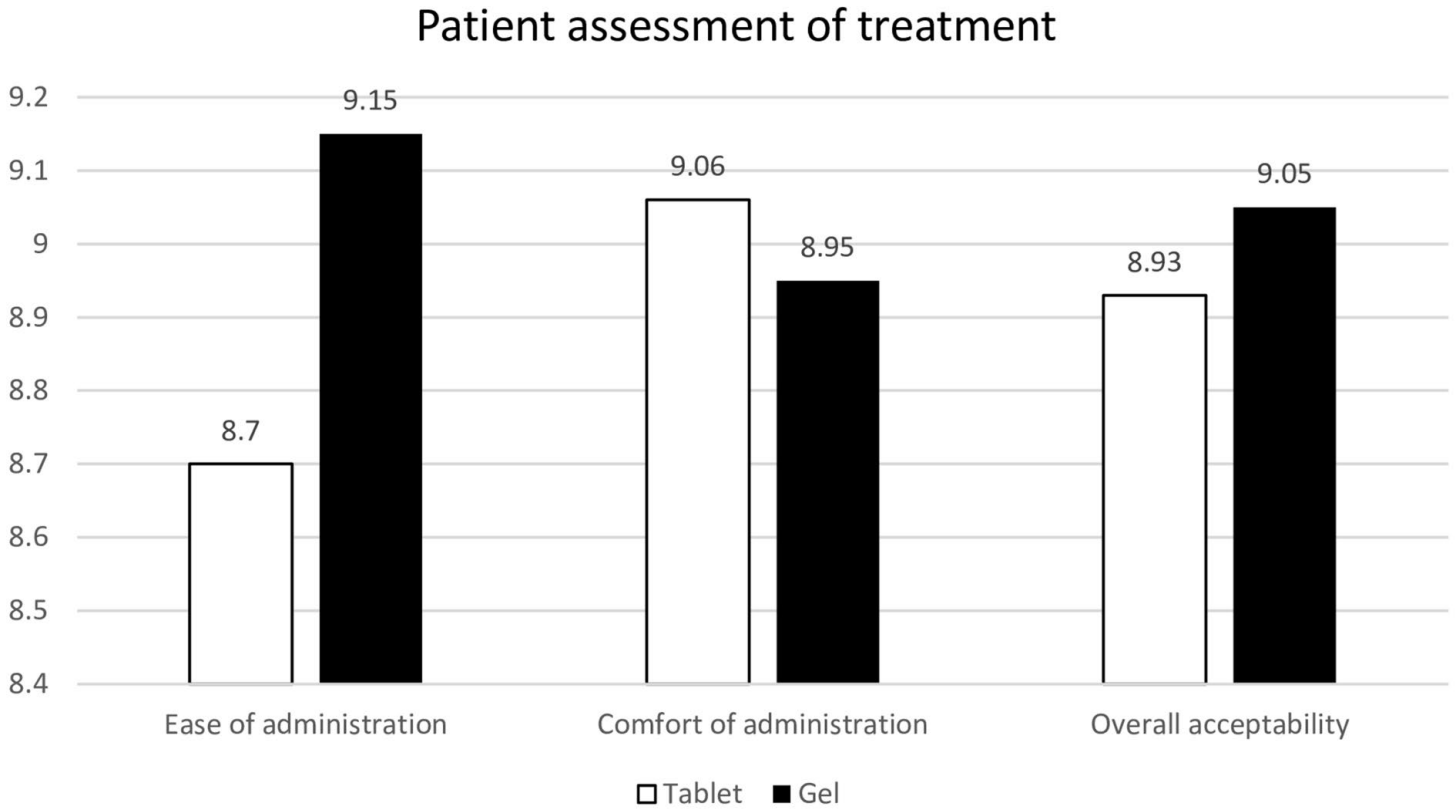
Patient assessment of treatment at week 24. Data are presented as mean with standard deviation. Comparisons were performed usin Student’s *t* test. *p* > 0.05 compared with baseline and between treatment groups

At the end of the trial, participants who had used both the tablet and gel forms rated ease, comfort, and acceptability similarly (Fig. 8). However, 55.3% expressed a preference for continuing the vaginal gel as their treatment of choice.

### Discussion

The severity of GSM symptoms guides treatment choices. According to the 2020 North American Menopause Society position statement [6], low-dose vaginal estrogen is an effective option for moderate to severe GSM. It relieves symptoms such as vaginal dryness and dyspareunia, lowers vaginal pH, and induces beneficial cytological changes, including increased superficial cells and decreased parabasal cells. When only genitourinary symptoms are present, local therapy is preferred over systemic therapy because of minimal systemic absorption.

This study demonstrated that a 10 µg estradiol hemihydrate vaginal tablet and a 10 µg vaginal gel both effectively improved postmenopausal vaginal atrophy after 12 weeks of treatment. The gel produced a significantly higher VMV than the tablet at 12 weeks (60.2 ± 12.0 vs 51.6 ± 23.8; P = 0.035, 95% CI 0.5 to 16.5), although the 95% CI crossed the noninferiority margin of 15. Per-protocol analysis showed means of 54.9 ± 21.0 for the tablet and 60.2 ± 12.0 for the gel (P = 0.161, 95% CI ‒2.1 to 12.6), with the 95% CI remaining within the noninferiority margin. This inconsistency suggests that the tablet could be noninferior either under optimal adherence conditions or if the dropout of three participants (all from the tablet group) had not occurred, as no participants were excluded in the gel group.

This study cannot definitively establish the noninferiority of the 10 µg estradiol hemihydrate tablet compared with the gel at 12 weeks. However, after the crossover, there was no further increase in VMV at 24 weeks in either group, and both exhibited identical VMV values. This result aligns with prior findings that improvements observed by 12 weeks are sustained for up to 52 weeks [20]. The benefits of vaginal estrogen continue as long as therapy is maintained.

At 12 weeks, the gel group had higher VHI scores and a significantly lower vaginal pH than the tablet group. The pH of K-Y Jelly (Johnson & Johnson, Skillman, NJ, USA) is approximately 3.5 [21], which may account for the more pronounced reduction in pH observed in the estradiol vaginal gel group. Its acidic nature and lubricating properties could enhance fluid volume during VHI assessment and decrease vaginal pH. Despite statistically significant differences in VMV, VHI, and pH, there were no discernible clinical differences, as the mean MBS severity decreased in both groups at 12 weeks compared to baseline, with no difference between them.

Numerous investigations have documented the favorable impact of vaginal estrogens on female sexual function and quality of life [22–24]. In the present study, both groups demonstrated a nonsignificant rise in total FSFI scores, but statistically significant improvements were observed only in the arousal, satisfaction, and pain domains. This is likely due to the multifaceted nature of sexual dysfunction in postmenopausal women, which involves both psychological and biological factors. While vaginal estrogen can address physiological symptoms, it may not alleviate underlying psychological issues. Additionally, only 35 study participants were sexually active, limiting the statistical power and generalizability of these results.

Endometrial thickness did not change significantly from baseline to week 12 in either the tablet or gel group, and it remained stable after the treatment switch until the study ended. This finding aligns with previous investigations [25]. A one-year endometrial safety study of a 10 µg estradiol vaginal tablet demonstrated no evidence of endometrial stimulation, hyperplasia, or cancer when evaluated via endometrial biopsies [26]. A Cochrane review similarly found no significant differences among various delivery methods regarding hyperplasia, endometrial thickness, or adverse events. [27] In line with the 2020 NAMS guidelines [6], these results suggest that routine endometrial surveillance is not warranted for asymptomatic women using low-dose vaginal estrogen.

No significant rises in serum estradiol were observed in either group at weeks 12 and 24, and levels remained comparable between the two groups and within the normal postmenopausal range [19]. This outcome is consistent with previous studies of the 10 µg estradiol vaginal tablet at 12 and 52 weeks [16, 26]. Although systemic absorption may occur more readily in atrophic vaginal epithelium, it diminishes as the epithelium matures [16]. Higher estradiol absorption tends to occur at the beginning of treatment, decreasing over time [26].

Compliance and patient satisfaction regarding ease, comfort, and overall acceptability were similar for both groups. This finding differs from previous studies by Rioux et al. [12] and Hosseinzadeh et al. [28] which reported higher patient acceptance of vaginal tablets over vaginal cream, possibly because the cream’s sticky consistency caused increased medication reflux and required sanitary protection. In contrast, the vaginal gel used in this study was formulated with K-Y Jelly, a hydrophilic gel containing hydroxyethylcellulose. This compound may reduce reflux because of its hydrophilic nature and the relatively small 0.8 mL volume administered. Consequently, participants did not report noticeable reflux with the gel.

This study has several strengths. First, it is the initial investigation comparing the efficacy of the two vaginal estrogen formulations available at Siriraj Hospital. Second, it employed a randomized design, with a single investigator evaluating all participants. Third, multiple instruments and parameters were used to assess both efficacy and safety, providing a comprehensive evaluation. Fourth, patient satisfaction data are likely accurate because the crossover design exposed each participant to both medications, allowing direct comparison within the same individual. Cost and availability are important considerations, especially in resource-limited settings. The estradiol gel, compounded from oral tablets, provided a significantly lower-cost alternative while maintaining clinical efficacy.

An important limitation is the absence of a washout period, which could have led to lingering effects from prior treatment. Consequently, the long-term outcomes at 24 weeks cannot be definitively attributed to each individual formulation, aside from patient satisfaction data. Other limitations of the study include its single-center design, which may affect generalizability, a relatively homogeneous participant population, and the short duration of each treatment period (12 weeks), which may be insufficient to assess long-term outcomes. Additionally, while VMV differences between formulations were statistically significant, the clinical relevance of this difference is uncertain, as both groups showed similar improvements in symptoms.

In conclusion, this study cannot definitively establish the noninferiority of the 10 µg estradiol hemihydrate vaginal tablet compared with the vaginal gel at 12 weeks. Nevertheless, both medications demonstrated clinically equivalent benefits for GSM, showed favorable safety profiles, and exhibited minimal systemic estradiol absorption. Patient satisfaction was comparable for both treatments, indicating that either option may be offered as a therapeutic choice. These findings provide valuable insight for guiding future treatment decisions.

## Data Availability

No datasets were generated or analysed during the current study.
